# Aktuelles zu neurogenen Dysfunktionen des unteren Harntraktes bei Multipler Sklerose

**DOI:** 10.1007/s00115-020-01046-0

**Published:** 2021-01-05

**Authors:** Burkhard Domurath, Peter Flachenecker, Thomas Henze, Wolfgang Feneberg, Anna Brandt, Ines Kurze, Ruth Kirschner-Hermanns, Albert Kaufmann, Jörn Bremer, Manuela Vonthien, Kerstin Ratering, Christoph Schäfer, Will Nelson Vance, Paul Schmidt

**Affiliations:** 1 Neuro-Urologisches Zentrum, Kliniken Beelitz GmbH, Paracelsusring 6A, 14547 Beelitz Heilstätten, Deutschland; 2 Neurologisches Rehabilitationszentrum Quellenhof, Bad Wildbad, Deutschland; 3 Praxis für Neurologie Dr. Blersch, Regensburg, Deutschland; 4 Marianne-Strauß-Klinik, Behandlungszentrum Kempfenhausen für Multiple Sklerose Kranke gemeinnützige GmbH, Berg, Deutschland; 5 Neurologisches Zentrum, Segeberger Kliniken, Bad Segeberg, Deutschland; 6 Querschnittgelähmten-Zentrum, Klinik für Paraplegiologie und Neuro-Urologie, Zentralklinik Bad Berka GmbH, Bad Berka, Deutschland; 7 Sektion Neurourologie, Klinik für Urologie und Kinderurologie, Universitätsklinikum Bonn und Neuro-Urologie, Neurologisches Rehabilitationszentrum der „Godeshöhe“ e. V., Bonn, Deutschland; 8 Zentrum für Kontinenz und Neuro-Urologie, Kliniken Maria Hilf GmbH, Mönchengladbach, Deutschland; 9 Querschnittgelähmtenzentrum, BDH Klinik Greifswald gGmbH, Greifswald, Deutschland; 10 Coloplast GmbH, Kühnstr. 75, 22045 Hamburg, Deutschland; 11 Rehaklinik für Neurologie und Orthopädie, Johanniter-Klinik am Romberg, Dortmund, Deutschland; 12 Statistik, Große Seestraße, 813086 Berlin, Deutschland

**Keywords:** Multiple Sklerose, Neurogene Störungen des unteren Harntrakts (NLUTS), Urodynamik, Harnblasenfunktionsstörungen, Assessments, Multiple sclerosis, Neurogenic lower urinary tract disorders (NLUTD), Urodynamics, Bladder dysfunction, Assessments

## Abstract

**Hintergrund:**

In der Routine steht man vor der Aufgabe, neurogene Störungen des unteren Harntraktes (NLUTD) bei Patienten mit Multipler Sklerose (MS) frühzeitig zu erkennen und adäquat zu therapieren. Verschiedene nationale Leitlinien geben dazu sehr unterschiedliche praktische Empfehlungen.

**Ziel der Arbeit:**

Erarbeitung eines einfachen, studienbasierten Algorithmus zum Nachweis von NLUTD bei Patienten mit MS, aus dem sich therapeutische Konsequenzen ableiten lassen.

**Material und Methode:**

Als direktes Ergebnis zweier multidisziplinärer Konferenzen wurde eine prospektive, multizentrische Studie initiiert. Deren Ziel war es, statistisch relevante Parameter für die Routinediagnostik von NLUTDs zu identifizieren. Als Goldstandard dienten Auffälligkeiten in der Urodynamik. In drei weiteren Konsensuskonferenzen wurden die Ergebnisse der Studie diskutiert, ein diagnostischer Algorithmus entwickelt und eine Erstlinientherapie konsentiert.

**Ergebnisse und Diskussion:**

Der vorgeschlagene Algorithmus ermöglichte das Erkennen einer NLUTD bei Patienten mit MS mithilfe von 4 statistisch signifikanten Prädiktoren: (1) dem Restharnvolumen, (2) der Anzahl der Harnwegsinfektionen (HWI) innerhalb der letzten 6 Monate, (3) der standardisierten Miktionsfrequenz und (4) dem Vorhandensein/Fehlen einer Harninkontinenz. Gestützt auf den Algorithmus benötigen ca. 75 % der Patienten keine urodynamische Untersuchung zur First-line-Therapieentscheidung. In 25 % der Fälle sind urodynamische Untersuchungen unerlässlich. Für die Routine notwendigen Assessments sind: die Anamneseerhebung, eine Restharnbestimmung, ein Miktionstagebuch und eine Uroflowmetrie (optional).

## Abstract (en)

**Background:**

In the clinical management of patients with multiple sclerosis (MS), the challenge is to make an early diagnosis and initiate adequate treatment of neurogenic disorders of the lower urinary tract (NLUTD). Various national guidelines provide practical recommendations which are sometimes discordant.

**Objective:**

To develop a simple evidence-based algorithm for detecting NLUTD in patients with MS that could be taken as a principle for deriving therapeutic consequences.

**Material and methods:**

A prospective multicenter study was initiated as a direct result of two multidisciplinary conferences. The aim was to identify statistically and clinically relevant parameters for the routine diagnosis of NLUTD in patients with MS. Urodynamic abnormalities served as the gold standard. At three subsequent consensus conferences, the results of the study were discussed, a diagnostic algorithm was developed and consensus was reached on a first-line treatment.

**Results and discussion:**

The proposed algorithm enables the detection of NLUTD in patients with MS with the help of four statistically significant predictors: 1) the residual urine volume, 2) the number of urinary tract infections (UTI) within the last 6 months, 3) the standardized micturition frequency and 4) the presence/absence of urinary incontinence. The newly developed algorithm has proved to be efficient with the following results: approximately 75% of the patients do not need a urodynamic examination for a first-line treatment decision. In 25% of cases, urodynamic examinations are essential for an adequate treatment decision. Routine assessments include the patient medical history, residual urine volume measurement, a micturition diary and a uroflowmetry (optional).

Die Diagnostik neurogener Funktionsstörungen des unteren Harntraktes (NLUTD) bei MS-Patienten ist eine besondere Herausforderung. In der Routine stellen sich folgende Fragen: 1. Wann sollten Patienten mit MS auf eine NLUTD hin untersucht werden? 2. Welche Patienten mit MS sollten gescreent werden? 3. Welche Screeningmethoden sind erforderlich? 4. Welche Screeningparameter geben Hinweise auf das Vorliegen einer NLUTD bei MS-Patienten? Eine multiprofessionelle Konsensusgruppe ging mit einer prospektiven Studie diesen Fragen nach und fand neue Antworten.

## Hintergrund und Fragestellung

Bei Diagnosestellung der MS treten Symptome einer neurogenen Harnblasenfunktionsstörung eher selten auf [5], 10 Jahre danach liegt deren Prävalenz bereits bei 80 % [27]. Manack et al. stellten bei der Analyse eines Kollektivs von 9315 MS-Patienten fest, dass das Bestehen einer NLUTD das Risiko für Krankenhauseinweisungen signifikant erhöht [21]. Die meisten Patienten berichten erst bei ihrer ersten urologischen Vorstellung, dass sie schon 10 Jahre unter Symptomen einer Blasenfunktionsstörung leiden [20]. Das bedeutet, dass den Patienten diese Lebensjahre verloren gehen, in denen sie eine deutlich eingeschränkte Lebensqualität infolge HWIs, Inkontinenz und Nierenkomplikationen in Kauf nehmen müssen, abgesehen von Verschlechterungen der MS durch diese Komplikationen [15, 18]. Mehr noch, über 56 % der Patienten mit urologischen Symptomen erhalten keine Behandlung dieser Beschwerden, wie ein aktueller Report des deutschen MS-Registers aufzeigt [9].

Um die Situation zu verbessern, geben spezielle nationale Leitlinien Empfehlungen für eine strukturierte Diagnostik einer neurogenen Störung des unteren Harntraktes (NLUTD) bei MS. Leider liegen die Empfehlungen über eine sinnvolle Diagnostik teilweise weit auseinander (Tab. 1).

**Table 1:** 

Kernpunkte		DEU [4]	ESP [22]	GBR [10]	FR [2]	USA [25]	ITA [11, 12]	BEL [24]	TUR [32]	Intern. [3]	CAN [19]
*Welche Patienten sollten auf NLUTD gescreent werden*	Asymptomatische Patienten	–	X	–	X	–	X	X	X	–	–
	Symptomatische Patienten	X	X	X	X	X	X	X	X	–	–
	Risikopatienten	–	–	–	–	–	–	–	–	X	X
*Eingeschlossene Screeningmethoden*	Miktionstagebuch	X	X	–	X	–	X	X	X	X	X
	Restharn	X	X	X	X	X	X	X	X	X	X
	Urinanalyse (Teststreifen)	X	X	X	X	X	X	X	X	X	X
	Urinkultur	Option	X	–	X	X	X	–	X	–	–
	Fragebogen zur Lebensqualität	–	X	–	X	–	X	X	–	X	0ption
	Ultraschall Blase, Niere	–	–	–	X	–	X	X	X	X	X
	Labor	Nierenfunktion	Nierenfunktion	–	HST, KREA	–	KREA	–	–	Nierenfunktion	KREA-Clearance
	Uroflowmetrie	Option	X	–	–	–	–	–	X	X	–
	Urodynamik	Option	X	–	X	X	X	X	–	X	X
*„Red flags“*	Alter (Jahre)	–	–	–	≥55	–	≥55	>50	–	–	–
	Geschlecht	–	–	–	♂	–	♂	♂	–	–	–
	EDSS	–	>3,0	<6,0 6,0–7,0 7,5–8,0 >8,0	≥6,0	–	≥3	<6,0 6,0–7,0 7,5–8,0 >8,0	–	Erhöht	Erhöht
	Dauer MS (Jahre)	–	–	–	–	–	>10	>15	–	–	–
	Restharn (ml)	–	≥150	>100	≥100	≥100	≥100 oder 1/3 Blasenkapazit	>100–150	Erhöht	Erhöht	Erhöht, >300 ml?
	Harnwegsinfekte/Jahr	–	≥3	–	≥3 oder mit Fieber	–	≥3	≥3	–	≥3	–
	Inkontinenz	X	X	X	–	X	X	X	–	X	–
	Häufige Miktionen	X	X	X	–	X	X	X	–	X	–
	Drang	X	X	X	–	X	X	X	–	X	–
	Erschwerte Miktion	X	X	–	–	X	X	X	–	X	–
	Auffälligkeit im Ultraschall	–	–	–	X	–	X	X	–	X	X
*Überweisung zu einem (Neuro‑)Urologen geregelt*		X	X	–	X	X	X	X	X	X	X

*DEU* Deutschland, *ESP* Spanien, *GBR* Großbritannien, *FR* Frankreich, *ITA* Italien, *BEL* Belgien, *TUR* Türkei, *CAN* Canada

Eine interprofessionelle Konsensusgruppe diskutierte diese Problematik und kam zu dem Schluss, die Relevanz der verschiedenen Parameter und der unterschiedlichen Expertenmeinungen im Rahmen einer multizentrischen Studie zu klären.

## Methoden

Zwei Konsensuskonferenzen dienten der Auswertung der Literatur zu NLUTDs bei Patienten mit MS, der Sammlung relevanter Parameter und der Konzipierung einer prospektiven Evaluationsstudie. Nach der Studie wurden die Ergebnisse in zwei weiteren Konferenzen ausgewertet, ein diagnostischer Algorithmus etabliert und statistisch überprüft. Im Rahmen eines 5. Konsensustreffens konnten daraufhin Empfehlungen zum Basisscreening, zur Erstlinientherapie und zur erweiterten Diagnostik erarbeitet werden.

### Studiendesign

Da urodynamisch nachweisbare NLUTDs bei MS-Patienten ohne urologische Symptome keine Seltenheit sind, wurden diese Patienten in die Studie aufgenommen [28]. Die Einschlusskriterien waren: Alter über 18 Jahre und eine nach den McDonald-Kriterien diagnostizierte MS mit den typischen Verlaufsformen RRMS – schubförmig remittierend MS, SPMS – sekundär progrediente MS und PPMS – primär progrediente MS [29]. Ausgeschlossen wurden nur Patienten mit einem Dauerkatheter oder mit einer urodynamisch abgeklärten NLUTD.

Als Zielgröße diente eine abnormale Blasenfunktion in der Urodynamik (Blasendruckmessung). Alle Patienten waren aufgefordert, eine Uroflowmetrie (Harnstrahlmessung) durchzuführen. In jedem Fall erfolgte eine Restharnbestimmung. Alle Patienten füllten einen Anamnesefragebogen aus und führten ein Miktionsprotokoll über 3 Tage. Da die Miktionsfrequenz mit dem Ausscheidungsvolumen pro Tag im Zusammenhang steht, erfolgte eine Standardisierung der Miktionsfrequenz auf eine einheitliche Ausscheidungsmenge von 2000 ml nach folgender Formel:

$$\text{Standardisierte Miktionsfrequenz}/24\mathrm{h}=\frac{\text{Anzahl Miktionen in}\,48\,\text{Stunden}\mathrm{x} 2000\mathrm{ml}}{\text{Ausscheidungsmenge in}\,48\,\text{Stunden}\,\left(\mathrm{ml}\right)}$$

Für die Auswertung des Miktionsprotokolls wurden 2 aussagefähige Tage (48 h) ausgewählt.

### Statistik

Um den Effekt verschiedener Parameter abzuschätzen, wurden logistische Regressionsmodelle herangezogen. Die Auswahl der relevanten Parameter erfolgte mithilfe einer schrittweisen Selektion der Variablen nach dem Akaike-Informationskriterium (AIC; [1]). Die Schwellenwerte der Prädiktoren wurden unter Verwendung einer rekursiven Partitionierung identifiziert [6]. Schlussendlich erfolgte die Einschätzung der prognostischen Qualität ausgewählter Klassifikatoren für die Vorhersage einer abnormalen Urodynamik mithilfe üblicher Standardleistungsmessungen für binäre Klassifikatoren (Sensitivität, Spezifität usw.).

## Ergebnisse

Insgesamt wurden 121 Patienten in die Evaluationsstudie eingeschlossen (Tab. 2).

**Table 2:** 

Parameter	Mittelwert ± Standardabweichung	Intervall
Alter (Jahre)	49,3 ± 11,3	19–75
Geschlecht	Männer: 33 Frauen: 88	–
EDSS	3,8 ± 2,1	0,5–8,0
MS-Dauer (Jahre)	12,2 ± 9,6	0–42
MS-Verlauf	RRMS – 56,8 % SPMS – 31,3 % PPMS – 11,9 %	–

Das Alter, das Geschlecht, die Dauer der MS und der EDSS wurden von der automatischen Variablenselektion via AIC als nicht relevant in Bezug auf eine NLUTD gewertet.

**Figure 1:**
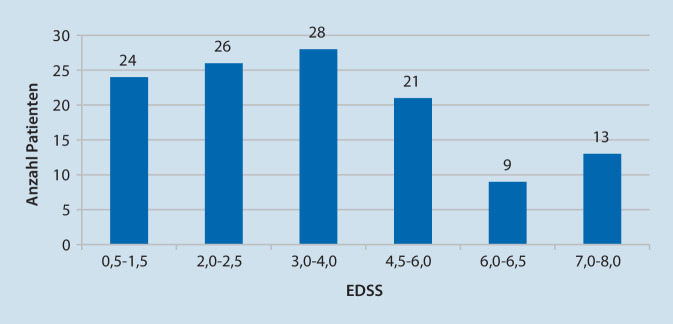

Die EDSS-Verteilungskurve zeigt die übliche Bimodalität (Abb. 1; [33]). Der EDSS-Median lag bei 3,5 und ist nahe am Median des MS-Register von 3,0 [9].

Als Prädiktoren einer gestörten Blasenfunktion (auffällige Urodynamik) konnten 4 Parameter mit den entsprechenden Odds Ratio (OR) und 95 %-Unsicherheitsintervallen (KI) ermittelt werden:

- standardisierte Miktionsfrequenz (OR = 1,24; KI:[1,07–1,49]),
- Rate an HWIs in den vorausgegangenen 6 Monaten (OR = 2,03; KI: [1,04–6,68]),
- Inkontinenz (OR = 3,93; KI:[1,17–15,7]) und
- Restharn (OR = 1,25; KI:[1,07–1,62]).

Für die 4 oben genannten Prädiktoren wurden statistische Grenzwerte ermittelt, bei denen sich urodynamische Auffälligkeiten mit hoher Sicherheit erwarten ließen (Tab. 3).

**Table 3:** 

Parameter	Unauffällig	Auffällig
Standardisierte Miktionsfrequenz	≤13	>13
Kontinenz	Kontinenz	Inkontinenz
HWI-Rate/6 Monate	0	>0
Restharn (ml)	<70	≥70

Die 4. Konsensuskonferenz entwickelte einen 3‑mal-4-Felder-Algorithmus. Die 3 Methodenzeilen wurden aus den Prädiktoren gebildet (Tab. 4).

**Table 4:** 

Methode	Prädiktoren	Gruppe 1	Gruppe 2	Gruppe 3	Gruppe 4
*Miktionstagebuch (3 Tage)*	*Standardisierte Miktionsfrequenz*	≤13	>13	Variabel	≤13
*Sonographie*	*Restharn (ml)*	<70	<70	≥70	<70
*Anamnese*	*HWI/6 Monate* *und/oder Harninkontinenz*	Keine	Ja oder nein	Ja oder nein	Ja

Wenn man die Grenzwerte der 4 Prädiktoren kombiniert, ergeben sich 16 mögliche Kombinationen (Cluster). Nach statistischer Überprüfung verblieben davon 4 Cluster, aus denen sich die 4 Gruppenspalten des Algorithmus ergaben (Tab. 4). Die Sensitivität bezüglich einer urodynamischen Auffälligkeit lag in diesem 3‑mal- 4-Algorithmus bei 95 %, der positive Voraussagewert bei 91 %.

Insgesamt zeigte sich ein geringer Anteil an Patienten mit einer unauffälligen Urodynamik (14 %) in unserem Studienkollektiv.

Der Gruppe 1 wurden Patienten zugeordnet, die die Grenzwerte der Prädiktoren nicht erreichten (keine urologischen Symptome). Zu erwarten wäre, dass sich in dieser Gruppe mit unauffälliger Symptomatik keine oder nur sehr wenige Fälle mit urodynamischen Auffälligkeiten finden lassen. In dieser Gruppe hatten aber nur 11 der 23 Patienten einen unauffälligen, 12 Patienten einen auffälligen urodynamischen Befund.

Patienten der Gruppe 2 (*n* = 23, 19,0 %) fielen vor allem durch eine erhöhte standardisierte Miktionsfrequenz auf, bei unauffälligem Restharn. Diese Patienten hatten alle urodynamische Auffälligkeiten.

Das Hauptmerkmal der größten Gruppe von Patienten in unserem Studienkollektiv (Gruppe 3, *n* = 38, 31,4 %) war eine Restharnmenge von ≥70 ml. Alle Patienten mit einer normalen Urodynamik hatten einen Restharn unter 70 ml. In der Gruppe 3 fanden sich somit nur Patienten mit einer auffälligen Urodynamik.

Patienten der Gruppe 4 (*n* = 33, 27,2 %) unterscheiden sich von denen der Gruppe 1 durch das Vorliegen einer Harninkontinenz und/oder von vorausgegangenen HWIs. Diese Gruppe ist urodynamisch sehr heterogen (15,2 % – normale Urodynamik, 20 % – Inkontinenz unterschiedlichen Typs, 27 % – Detrusorunteraktivität, 37,5 % – Detrusorüberaktivität, 20 % – veränderter Harndrang und 40 % – Detrusor-Sphinkter-Dyssynergie).

## Diskussion

Die vorliegende Studie bestätigt, dass der Anteil urodynamisch feststellbarer NLUTDs bei Patienten mit MS hoch ist. In unserer Studie hatten 86,0 % aller Patienten eine auffällige Urodynamik. Dieser hohe Prozentsatz ist vergleichbar mit dem bei Ineichen et al. – 86,5 % und bei Wiedemann et al. – 78,0 %, auch wenn man berücksichtigt, dass sich die Einschlusskriterien dieser Studien von denen unserer Studie unterscheiden [17, 31]. Die von uns ursprünglich ins Auge gefasste risikoadaptierte Gruppenbildung im primär entworfenen Algorithmus (niedriges, mittleres, hohes Risiko für eine abnormale Urodynamik) musste deshalb aufgegeben werden. Sogar Patienten ohne urologische Symptome hatten in 52,2 % der Fälle auffällige urodynamische Befunde, sodass der Begriff „niedriges Risiko“ nicht zutraf. Der Anteil auffälliger Urodynamiken in den Gruppen mit einem angenommenen mittleren und hohen Risiko war so hoch und lag in beiden Gruppen so nahe beieinander (84,6 % und 92,6 %), dass sich deren Prognosegüte nicht mehr voneinander unterschied. Als weiteres Problem wurden die aus der Literatur entnommenen „red flags“ identifiziert, die nicht validiert waren (Tab. 1). Deshalb erfolgte eine Umorientierung auf die statistische Bestätigung von Parametern als Prädiktoren und auf deren statistisch ermittelte Grenzwerte. Das erlaubte eine Umstellung auf einen therapeutisch orientierten Algorithmus nach statistisch validierten Diagnosekriterien.

Entgegen der Ausführungen mancher Leitlinien (Tab. 1) konnten wir die Dauer der MS, das Alter, das Geschlecht und den MS-Verlauf nicht als Prädiktoren einer NLUTD identifizieren. Wir bestätigen damit die Ergebnisse anderer Studien. Auch der EDSS war für die Vorhersage einer NLUTD ungeeignet (OR = 0,91; KI :[0,69–1,2]). Ineichen et al. fanden bei Patienten mit MS einen EDSS-Cut-off von 5,0 für das Risiko einer Schädigung des oberen Harntraktes [17]. Dieses theoretische Risiko für Patienten mit MS definierten Ineichen et al. nach Kriterien, die ursprünglich für querschnittgelähmte Patienten ermittelt worden waren. Sie ermittelten in 87 % ein theoretisches Risiko für eine Schädigung des oberen Harntraktes bei MS-Patienten. Eine tatsächliche Nierenfunktionsschädigung ist aber bei Patienten mit MS im Gegensatz zu querschnittgelähmten Patienten ein Ereignis im eher niedrigen einstelligen Prozentbereich [26, 27]. Giannantoni et al. stellten eine Korrelation zwischen dem EDSS und einer Detrusorunteraktivität fest, wobei nur 15,4 % der Patienten mit einer auffälligen Urodynamik eine Unteraktivität aufwiesen [13]. Wiedemann et al. fanden einen EDSS von >6,5 (Rollstuhlabhängigkeit) als Prädiktor einer NLUTD [31]. Dieser Prädiktor aber ist sehr schwach, da er nur 14,0 % der Patienten mit einer auffälligen Urodynamik identifizierte. In unserer Studie wären es nur 11,4 %. Wir halten deshalb eine EDSS-basierte Risikostratifizierung, wie in einigen Leitlinien vorgeschlagen (Tab. 1), für wenig Erfolg versprechend.

Im Jahr 2012 bestätigten Tadayyon et al. frühere Untersuchungen über einen hohen Anteil an urodynamischen Auffälligkeiten bei MS-Patienten, die keine urologischen Beschwerden angaben (62 %; [13]). In unserer Studie waren es in der Gruppe 1 52,1 %. Tadayyon et al. fanden im Verlauf, dass 94 % dieser Patienten innerhalb eines Jahres urologische Symptome entwickelten. Einige nationale Leitlinien empfehlen ein Basisscreening für Patienten, die keine urologischen Symptome angeben (Tab. 1). Unsere Ergebnisse untermauern diese Empfehlung. Um Patienten mit einer verdeckten NLUTD herauszufinden, kann eine Uroflowmetrie statt einer Urodynamik eingesetzt werden. Wir bestimmten die OR der Uroflowmetrie bezüglich einer auffälligen Urodynamik in unserer Studie mit 4,80 (KI: [1,4–19,21]), die Sensitivität mit 79,3 % und die PPV mit 94,0 %. Symptomlose Patienten mit auffälligem Uroflow benötigen nach unserer Meinung keine Therapie, sie sollten aber in kürzeren Abständen kontrolliert werden (Konsensusempfehlung: nach 6 Monaten, Tab. 5). Beim Rescreening geht es darum, ob die Grenzwerte der Prädiktoren überschritten werden.

**Table 5:** 

	Gruppe 1	Gruppe 2	Gruppe 3	Gruppe 4
*Erweiterte Diagnostik im Rahmen des Basisscreenings*	Uroflowmetrie	Keine	Keine	Urodynamik!
*Erstlinientherapie*	Keine	Antimuskarinika (cave: Restharn)	ISK, evtl. Antimuskarinika	Keine

Kennzeichnend für die 2. Gruppe ist eine erhöhte Miktionsfrequenz (≥13-mal/24 h), deren Standardisierung für die Anwendung im Algorithmus entscheidend ist. Die nichtstandardisierte Miktionsfrequenz schwankte von 4‑mal/24 bis 23-mal/24 h bei Ausscheidungsmengen zwischen 600 und 3000 ml. Nach Haggiag et al. taugt die nichtstandardisierte Miktionsfrequenz mit einer Sensitivität von nur 26,3 % nicht als Prädiktor einer gestörten Harnblasenfunktion [14]. Die standardisierte Miktionsfrequenz korrelierte in unserer Studie gut mit einer Detrusorüberaktivität (OR = 1,16; KI:[1,07–2,06]), sodass „first-line“ eine orale oder transdermale detrusordämpfende Therapie mit Antimuskarinika gerechtfertigt ist (Tab. 5; [16]).

In allen Leitlinien wird bei einem erhöhten Restharn der intermittierende Selbstkatheterismus (ISK) empfohlen, wobei uneinheitliche Grenzwerte eines pathologischen Restharns festgelegt wurden (Tab. 1). Unser Grenzwert von 70 ml Restharn in der Gruppe 3 ist relativ niedrig, ähnelt aber dem in der kleinen Studie von Tracy et al. [30]. Der Cut-off bei 70 ml dient primär dem Aufdecken einer NLUTD bei MS-Patienten. Die Zweckmäßigkeit und Durchführbarkeit des ISK muss im Einzelfall geprüft werden und kann entsprechend der 5. Konsensuskonferenz nicht allein an einer konkreten Höhe des Restharns festgemacht werden (Gruppe 3, Tab. 5). Ein ISK setzt ausreichende kognitive, visuelle und sensomotorische Fähigkeiten voraus, die bei MS-Patienten nicht immer gegeben sind. In der 3. Gruppe korrelierte die Restharnmenge nicht mit der HWI-Rate, wie bereits aus anderen Studien bekannt [7]. Eine Patientin mit einem Restharn von 100 ml hatte 8 HWIs in den letzten 6 Monaten. In der Gruppe mit Restharnmengen zwischen 200 und 300 ml hatte keiner der Patienten einen HWI (*n* = 9). Für eine individuelle Handhabung des Katheterismus spricht auch die Tatsache, dass der Anteil von Patienten mit Detrusorüberaktivität in der 3. Gruppe relativ hoch ist (59,5 %), bei einem Miktionsfrequenzmedian von 16-mal/24 h und einem Restharnmedian von 172 ml. Bei der Therapieentscheidung für ein den Detrusor dämpfendes Medikament ist mit der Zunahme der Restharnmenge zu rechnen. Ein Urologe sollte frühzeitig einbezogen werden.

Die Resultate unserer Studie ergaben, dass Patienten der Gruppe 4 (*n* = 33, 27,3 %) sehr unterschiedliche urodynamische Befunde bieten. Deshalb fehlt bei diesen Patienten ein First-line-Therapieansatz. Vor einer Therapieentscheidung halten wir deshalb eine urodynamische Abklärung bei jedem Patienten dieser Gruppe für sinnvoll (Tab. 5). Zum Beispiel waren 67,5 % der Patienten der 4. Gruppe inkontinent, von diesen hatten aber nur 26 % eine Detrusorüberaktivität. Das verdeutlicht, dass eine antimuskarine Therapie zur Behebung der Inkontinenz bei Patienten der Gruppe 4 wenig Aussicht auf Erfolg hat. Die aktuellen Leitlinien des National Institutes for Health and Care Excellence (NICE) empfehlen, bei Patienten mit MS keine urodynamischen Untersuchungen zu veranlassen [23]. Dieser Argumentation kann aus unserer Sicht nicht gefolgt werden. Die meisten Leitlinien sehen das ebenso (Tab. 1).

Zusammenfassend zeigen die Ergebnisse dieser Studie, dass eine NLUTD bei Patienten mit MS mit relativ geringem Aufwand sicher diagnostiziert werden kann, ohne dass dazu eine invasive Urodynamik erforderlich ist. Eine First-line-Therapie kann nach Zuordnung zu den einzelnen Gruppen aus dem vorgeschlagenen Algorithmus abgeleitet werden. Es empfiehlt sich aber, eine enge Beziehung zu einem niedergelassenen Urologen aufzubauen, der wünschenswert eine Uroflowmetrie vorhält. Die meisten Querschnittzentren und einige spezialisierte Rehabilitationszentren verfügen über eine qualifizierte Neurourologie. Sie sind zwar im Allgemeinen nicht wohnortnah gelegen, angesichts deren Expertise bei neurourologischen Fragestellungen sollte man aber den Kontakt zu diesen Zentren auch bei MS suchen [8].

### Limitationen

- Eine urodynamisch nachweisbare NULTD war bei den Patienten in unserer Studie häufig nachzuweisen. Um die Aussagekraft des Algorithmus zu verfeinern, sind mehr Patienten mit unauffälliger Urodynamik erforderlich.
- Die Gruppe der Patienten, die keine urologischen Symptome angab, war relativ klein, sodass wegen der praktischen Bedeutung eine Bestätigung unserer Ergebnisse in einer größeren Studie wünschenswert wäre.
- Unsere Studie bestätigt die diagnostische Relevanz eines Restharns ab 70 ml bei Patienten mit MS. Die Bestimmung eines therapeutisch relevanten Restharngrenzwertes im Kontext mit anderen klinischen Parametern müsste in zukünftigen Studien geklärt werden.
- Die aus dem Algorithmus abgeleiteten Therapiemaßnahmen sind im Expertenkonsensus entstanden und bislang nicht auf ihren Erfolg hin in der Praxis evaluiert worden.

## Fazit für die Praxis

- Vor jedem Screening sollte ein akuter HWI ausgeschlossen werden, da die Symptome eines HWI den Symptomen einer NLUTD bei MS-Patienten sehr ähnlich sind.
- Ein Patientenfragebogen und ein Miktionsprotokoll bilden das diagnostische Grundgerüst, um NLUTDs bei Patienten mit MS frühzeitig erkennen zu können.
- Eine Restharnbestimmung ist unverzichtbar.
- Die Schwellenwerte für die standardisierte Miktionsfrequenz (>13-mal/24 h), für den Restharn (≥70 ml) sowie für die Rate von HWIs (>0-mal/6 Monate) und einer Harninkontinenz (ja/nein) erlauben eine Einordnung in einen vorgeschlagenen Algorithmus und eine Erstlinientherapie.
- Patienten ohne festzustellende urologische Auffälligkeiten weisen in über 50 % eine neurogene Dysfunktion des unteren Harntraktes auf und sollten einer Uroflowmetrie zugeführt werden, um eine verdeckte NLUTD nichtinvasiv erkennen zu können.
- Eine urodynamische Untersuchung muss für bestimmte Patienten vor einer sinnvollen Therapieentscheidung im Behandlungskonzept berücksichtigt werden.
